# Chemicals from Food Supply Chain By-Products and Waste Streams

**DOI:** 10.3390/molecules24050978

**Published:** 2019-03-11

**Authors:** Marta Coma, Afroditi Chatzifragkou

**Affiliations:** 1 Centre for Sustainable Chemical Technologies (CSCT), University of Bath, Claverton Down, Bath BA2 7AY, UK; 2 Department of Food and Nutritional Sciences, The University of Reading, P.O. Box 226, Whiteknights, Reading RG6 6AP, UK

Circular economy and bioeconomy concepts have been introduced within an EU framework to sustainably overcome the dominant development model of “take, make, and dispose”, which has contributed to current economic, environmental, and societal burdens. Although the circular economy focuses on enhancing the efficiency of resource utilisation, bioeconomy relies on the conversion of renewable carbon reserves, derived from organic wastes as well as agricultural or forestry biomass, into diversified end-products and materials [1]. Therefore, the organic streams, which are generated along the food supply chain and are not aimed for human consumption, are an excellent feedstock to utilise within a circular bioeconomy. This Special Issue presents a series of studies that evaluate the potential recovery of targeted molecules from the food waste supply chain as well as enhancements to current technologies.

Biobased feedstocks are the alternative source for future energy, chemicals and materials. Not only because petroleum-based feedstocks are a finite resource, but because of their environmental burden and their need to be functionalized with, for example, oxygen and nitrogen, which are already present in biomass [2]. Every year, 1.3 billion tones of municipal solid waste (MSW) are accumulated globally, a number projected to expand to 2.2 billion tones by 2025. Waste should be viewed as a bioresource; such a perspective would ease its management, which is currently based on landfilling and incineration, with only a 38% being recovered. The organic portion of MSW is approximately 46% *w*/*t*; its current management within developing countries consists of animal feed, compost, anaerobic digestion, incineration, and disposal in landfills. Through enhanced resource efficiency, feedstock requirements for European industries could be reduced up to 24% by 2030, a figure achievable along the value chains [1].

Food supply chain waste (FSCW) is generated throughout the life cycle of different food supply chains and is classified according to three types: farm losses/waste (during agricultural production and harvesting), postharvest losses/waste (during postharvest handling and storage, manufacturing, distribution, and retailing), and consumer waste (both within the household and out of the home) [3]. For example, out of a total of 10 million tones in the UK food supply chain, food manufacturers waste 17% of food yearly, with 28% of food by-products being sent to animal feeding or rendering (6%) and 7% of food surplus redistributed or sent to animal feeding. Accordingly, only 48% of the food supply chain reaches its destination without losing economic value [4]. Furthermore, in medium/high-income countries, household food waste makes up the largest share in total FSCW. In the EU, approximately 45 million tones or 45% of the total food losses and waste was found at the household level [3].

Biorefinery concepts could be a core element for the exploitation of biomass/waste towards the manufacture of marketable intermediates and end-products [1], being the variability of food waste quantities the key factor for process design. It would be beneficial to extract valuable compounds from uneatable waste for valorisation processes, if it was possible, to manufacture new products for human consumption; however, the influence of the state of the feedstock (e.g., spoilage level) on the quality and quantity of the extracted materials must be assessed [4]. The recovery of nutritional, functional, and textural properties from the food supply chain waste will be an important future driver. For example, new food industry additives could be derived from protease inhibitors (appetite suppressants) from potato or essential oils, flavonoids, and pectin from citrus waste or other bioactive compounds including polyphenols, carotenoids, vitamins, antioxidants, flavonoids, and fibers extracted from vegetable and fruits waste [1,2]. In this Special Issue, two studies are presented in relation to the potential recovery of nutraceuticals from vegetable waste [5,6].

Liu et al. [5] investigated the phytonutrient concentrations of broccoli to determine that stem and leaf by-products, which make up nearly 70% of the weight of the plant, could be used as a source of valuable nutrients. Although most of the amino acids and minerals such as Fe, Zn, and P are found in the floret fraction, stems contain most of the sugars (fructose, glucose, sucrose, and maltose). Broccoli leaves contain a high concentration of calcium, manganese, carotenoids, chlorophylls, vitamins E and K, as well as total phenolic content, all known by their antioxidant activity. Broccoli leaves also presented the highest myrosinase activity, which might translate on the generation of potent anticancer compounds. Therefore, these results might support future consumption of broccoli agrowaste as well as the extraction of its nutraceutical compounds.

The recovery of carotenoids from fruit and vegetable wastes by supercritical fluid extraction (SFE) was studied in De Andrade Lima et al. [6]. Fibre content (both type and concentration) on vegetable and fruit matrices is critical because it may render the extraction of targeted molecules through SFE challenging by limiting their dissolution into the solvent phase. A mixed sample of 14 vegetable samples was employed to simulate an applicable and economically feasible industrial scenario in which SFE waste extraction within an industrial establishment/processor recovers and exploits value-added components from multiple vegetable- and fruit-derived by-products and waste streams. The SFE process was able to extract carotenoids from a mixed fruit and vegetable matrix, with high recovery levels (74% to 99% *w*/*w*), validating a previously developed SFE–CO_2_ model for optimum recovery of carotenoids.

The composition of the organic fraction of MSW widely differs according to the place and time of collection; in contrast, the composition of FSCW is dependent on the nature of the original raw materials. Although biosurfactants and biopesticides are produced from food waste fermentation, the most common microbial bioconversions relate to the production of bioethanol [1]. Unlike other commodity flows, food is a biological material subject to degradation, and different food stuffs have different and variable nutritional values [7]. Furthermore, as a result of the high moisture content of the organic fraction of MSW, incineration is highly energy-intensive; concurrently, landfilling poses numerous pollution issues and its direct application as animal feed bears significant risk with regard to the propagation of diseases [8]. Therefore, anaerobic digestion (AD) is the preferred treatment route for food waste because its multiple steps and microorganisms can degrade different types of biomass into biogas.

Although AD is a reliable technology for converting waste to energy, an increase in methane concentration could facilitate its co-use with natural gas. However, the low economic performance of the process, due to low methane content, may incur limitations, particularly in terms of the biodegradability and inhibition of substrates, given the presence of substances with antimicrobial actions such as d-limonene [4,8]. Approaches to enhance the methane content in AD, such as co-digestion or a two-step AD process, are discussed in this Special Issue.

Pilarska et al. [9] evaluated three types of confectionary waste, comprising chocolate bars, wafers, and filled wafers, for biogas production. All three substrates resulted suitable for AD with a high content of biogas being produced (on the range of 60–73%). Although all substrates presented yields between 70% to 90% of the theoretical potential, higher efficiencies were achieved for the filled wafers and lower efficiencies for the wafers. Chocolate bars presented a higher acidification and thus lower pH and higher volatile fatty acids accumulation, which resulted in lower than expected efficiencies due to substrate composition. The study also proposed an estimation of theoretical potentials on the basis of substrate chemical composition. Although confectionary waste seemed to have a high biodegradability, enhancing methane production, MSW as a substrate for AD is known by its low methane production. This is mainly due to the lower biodegradability of the organic fraction of MSW in comparison with food waste as well as its high solids concentration, which usually require a longer hydrolysis time. To enhance biogas production, Seruga et al. [10] investigated the effect of the introduction of co-substrates to increase methane production while stabilizing the fatty acids accumulation on a thermophilic AD process to enhance the hydrolysis of solids. The organic fraction of MSW was obtained from a full-scale mechanical biological treatment and was co-digested with restaurant waste, corn whole stillage, or effluents from the cleaning of chocolate transportation tanks, all of which were by-products or wastes from the food supply chain. Additions of 5–15% of co-substrates increased the biogas yield by 20–31%. Co-digestion also allowed the treatment of substrates with high organic concentrations, such as restaurant waste, which usually presents overloading issues in AD with the accumulation of fatty acids and process destabilization. Co-digestion can be also applied to reduce the concentration of inhibitory compounds in AD, such as d-limonene from citrus waste [4]. As an alternative, Lukitawesa et al. [11] presented a two-step AD process to treat citrus waste. The study aimed to investigate the effect of recirculation from the second to the first step of biogas production. Citrus waste was primarly digested in a stirred tank reactor; the liquid fraction was then fed into an up-flow anaerobic sludge bed. When recirculation was applied, methane yields doubled on average, solids reduction was 33–35% higher, and the reactor performance was more stable. Recirculation helped to maintain the pH around 5–6 during the first step, stabilizing the hydrolysis-acidogenic process, despite the presence of d-limonene, and enhancing overall methane production.

Interest in fuel and chemical production from lignocellulosic biomass has increased as an alternative to food crop feedstocks within biorefineries. By-product streams, such as those from food and drink production or food waste from households and supermarkets, are currently under investigation as renewable and more sustainable feedstocks. When easily fermentable sugars are extracted from raw material, the remaining substrates, such as proteins, lipids, cellulose, and hemicellulose, require a hydrolysis process that usually liberates a mixture of sugars and other compounds. Current research has focused on either studying organisms that can co-utilise these mixtures or controlling the specificity of the bioprocess. For example, Patel et al. [12] investigated the production of lipids from brewers’ spent grains as an oil substitute for biodiesel production. An organosolv- or microwave-assisted alkaline pretreatment was evaluated to release insoluble carbohydrates, which was later used by the oleaginous yeast *Rhodosporidium toruloides*, capable of co-utilising xylose and glucose. The organosolv pretreatment, together with an optimized C/N ratio of 500 during cultivation, achieved a product with more than 56% of lipid content. The profile of the biodiesel obtained also satisfied the criteria for use as a transportation fuel. In contrast, De Groof et al. [13] reviewed the operational conditions necessary for a mixed culture fermentation to produce medium-chain carboxylic acids (MCCA), that can be used as antibiotic replacements in animal feed or further processed into bulk fuels or solvents. Contrary to single-culture processes, these systems allow the treatment of more complex and variable substrates as food waste; however, their yields, selectivity, and, accordingly, economical viability, become more challenging to control. The review states that the production of MCCA is enhanced when substrates contain or easily produce ethanol or lactic acid, which are intermediate substrates; furthermore, mesophilic temperatures, an acid to neutral pH (5–7) and a higher substrate–inoculum ratio in comparison with AD (F/M > 5) are required. In addition, the process is enhanced with high loading rates, which benefits concentrated substrates that cannot be treated by AD.

Sarris et al. [14] investigated the feasibility of blending olive mill wastewaters (OMW) with crude glycerol as a substrate for the microbial production of added value compounds. OMW has a dark color, strong odor, and (phyto-)toxic properties, mainly attributable to its high quantities of phenolic compounds; it is also characterized by remarkably high biological (BOD) and chemical oxygen demand (COD). To this end, waste bioremediation was also targeted through the reduction of color and phenolics concentration of OMW. Partial or total substitution of water with OMW could support microbial growth and citric acid production in the yeast *Yarrowia lipolytica* ACA–DC 5029; however, a 30% color reduction and 10% phenolics reduction was also achieved. Moreover, in high glycerol media (170 g/L), the yeast produced 79 g/L of citric acid and 65.8 g/L of erythritol [14].

## References

[1] Maina S., Kachrimanidou V., Koutinas A. (2017). A roadmap towards a circular and sustainable bioeconomy through waste valorization. Curr. Opin. Green Sustain. Chem..

[2] Matharu A.S., de Melo E.M., Houghton J.A. (2016). Opportunity for high value-added chemicals from food supply chain wastes. Bioresour. Technol..

[3] Xue L., Liu G., Parfitt J., Liu X., Van Herpen E., Stenmarck Å., O’Connor C., Östergren K., Cheng S. (2017). Missing food, missing data? A critical review of global food losses and food waste data. Environ. Sci. Technol..

[4] Garcia-Garcia G., Stone J., Rahimifard S. (2019). Opportunities for waste valorisation in the food industry–A case study with four UK food manufacturers. J. Clean. Prod..

[5] Liu M., Zhang L., Ser S., Cumming J., Ku K. (2018). Comparative Phytonutrient Analysis of Broccoli By-Products: The Potentials for Broccoli By-Product Utilization. Molecules.

[6] De Andrade Lima M., Kestekoglou I., Charalampopoulos D., Chatzifragkou A. (2019). Supercritical Fluid Extraction of Carotenoids from Vegetable Waste Matrices. Molecules.

[7] Parfitt J., Barthel M., Macnaughton S. (2010). Food waste within food supply chains: Quantification and potential for change to 2050. Philos. Trans. R. Soc. B Boil. Sci..

[8] Zhang C., Su H., Baeyens J., Tan T. (2014). Reviewing the anaerobic digestion of food waste for biogas production. Renew. Sustain. Energy Rev..

[9] Pilarska A., Pilarski K., Wolna-Maruwka A., Boniecki P., Zaborowicz M. (2019). Use of Confectionery Waste in Biogas Production by the Anaerobic Digestion Process. Molecules.

[10] Seruga P., Krzywonos M., Wilk M. (2018). Thermophilic Co-Digestion of the Organic Fraction of Municipal Solid Wastes—The Influence of Food Industry Wastes Addition on Biogas Production in Full-Scale Operation. Molecules.

[11] Lukitawesa, Wikandari R., Millati R., Taherzadeh M., Niklasson C. (2018). Effect of Effluent Recirculation on Biogas Production Using Two-Stage Anaerobic Digestion of Citrus Waste. Molecules.

[12] Patel A., Mikes F., Bühler S., Matsakas L. (2018). Valorization of Brewers’ Spent Grain for the Production of Lipids by Oleaginous Yeast. Molecules.

[13] De Groof V., Coma M., Arnot T., Leak D., Lanham A. (2019). Medium Chain Carboxylic Acids from Complex Organic Feedstocks by Mixed Culture Fermentation. Molecules.

[14] Sarris D., Rapti A., Papafotis N., Koutinas A., Papanikolaou S. (2019). Production of Added-Value Chemical Compounds through Bioconversions of Olive-Mill Wastewaters Blended with Crude Glycerol by a Yarrowia lipolytica Strain. Molecules.

